# Clinical, humanistic, and economic burden of sickle cell disease in The Jazan Region, Saudi Arabia

**DOI:** 10.1371/journal.pone.0348759

**Published:** 2026-05-14

**Authors:** Abdulkarim M. Meraya, Otilia J. F. Banji, Santhosh Joseph Menachery, Hafiz Mosa Ali Malhan, Hatim Ali Asiri Halawi, Dhaifallah Moraya, Hamad Ahmed Hakami, Abdulaziz Mohammed Kubayni, Yunus Yahya Alfaifi, Hafiz Ibrahim Ghareeb, Mohammed Ali Abu Rasayn, Rayan Ahmed Alfaifi

**Affiliations:** 1 Pharmacy Practice Research Unit, Department of Pharmacy Practice, College of Pharmacy, Jazan University, Saudi Arabia; 2 Hematology Department, Mohammed Bin Nasser Hospital, Jazan, Saudi Arabia; 3 Hematology/ Oncology and Stem Cell Transplant, Prince Mohammed Bin Nasser Hospital, Jazan, Saudi Arabia; 4 Specialist Hospital, Jazan, Saudi Arabia; 5 College of Pharmacy, Jazan University, Jazan, Saudi Arabia; Versiti Blood Research Institute, UNITED STATES OF AMERICA

## Abstract

**Background:**

Sickle cell disease (SCD) constitutes a significant public health concern in the Kingdom of Saudi Arabia (KSA), affecting a substantial proportion of the population in the southern province of Jazan. The chronic and progressively incapacitating nature of SCD exerts a humanistic, psychological, and economic burden on the affected individuals and families. This study aims to assess this multifaceted burden on individuals with SCD in the Jazan region of KSA.

**Methods:**

We conducted a cross-sectional observational study using a self-administered validated questionnaire among SCD patients seeking medical services in various hospitals in the Jazan region. The questionnaire included sections to assess physical health, pain, social life, psychological distress, and financial burden. Descriptive statistics summarised participant characteristics. Comparative analyses using Chi-square tests and T-tests were conducted to explore differences in demographic variables between respondents reporting higher versus lower spending burdens. These analyses were intended to describe observed differences within the study sample rather than to infer population-level associations.

**Results:**

We included 110 individuals with SCD, with a mean age of 28.43 ± 9.66 years, 55.5% male, 4.5% non-Saudi, single (62.7%), with a little less than half residing in cities. The study showed low employment rates (36.4%) and high out-of-pocket expenditures for over half the patients, with transportation being the largest expense (SAR 828.51 ± 925.84/USD 220.94 ± 246.89 per month). High spending burden was associated with poorer physical health (*p* < 0.001). Hydroxyurea was the most commonly used therapeutic option (81.8%), while the use of newer agents, such as L-glutamine and crizanlizumab, was limited. Psychological distress was significantly associated with age (*p* = 0.008) and marital status (*p* = 0.001), with married individuals being more distressed. Social scores (*p* = 0.006) were significantly lower in individuals experiencing distress, while physical health (*p* < 0.001) and pain scores were higher (*p* < 0.001). Most reported disruption in social interactions and work life, compounded by a lack of family support. Furthermore, a majority felt their physicians lacked empathy and did not discuss treatment goals, necessitating half to avoid visits due to fear.

**Conclusion:**

SCD patients face considerable socioeconomic, psychological, and healthcare challenges, with out-of-pocket costs disproportionately burdening lower-income families. Individuals with SCD reported psychological distress, social disruption and reduced quality of life. An apparent variability in professional and familial support was noted, which may have contributed to differences in health care utilization. Additionally, self-reported quality of physician-patient communication and perceived empathy were identified as potential influences for their care-seeking behaviour. These findings reinforce the need for a larger-scale, comprehensive evaluation to confirm them and assess the need for interventions to improve healthcare access and address the psychosocial and economic burdens associated with SCD.

## Introduction

Sickle cell disease (SCD) is a monogenic hemoglobinopathy initiated by a point mutation in the beta-globin gene, leading to the fabrication of abnormal haemoglobin S (HbS) [1]. Under deoxygenation, HbS polymerizes, causing erythrocytes to sickle. These malformed, rigid cells activate a pathophysiological cascade characterized by chronic haemolytic anaemia, vaso-occlusion, ischemia-reperfusion injury, and a state of chronic inflammation [2,3]. This process is clinically established as a high-burden, multi-system disorder with complications comprising intermittent vaso-occlusive crises (VOC), predisposition to severe infections, and progressive organ damage [4].

Globally, SCD poses a significant health burden, with an estimated 300,000 children born with the disease annually [5]. Within the Kingdom of Saudi Arabia (KSA), SCD is a major public health concern, with a historically high incidence in the Eastern and South-western provinces [6]. A recent large-scale, cross-sectional study by Alhawiti et al. (2025) reported a national prevalence of 3.2% among Saudi residents, acknowledging the condition’s sizeable footprint and emphasizing meaningful associations with comorbidities like heart disease, hypertension, and diabetes [7].

The clinical burden of SCD is intense. VOC, the hallmark of the disease, results in acute-severe pain and is a primary cause of emergency department visits and hospitalizations [4]. Acute and chronic complications, such as VOC and stroke, contribute to significant morbidity and reduced life expectancy. Additionally, the effect of SCD extends beyond the usually recognized complications to affect multiple organ systems. Remarkably, cardiac association is a key concern; a systematic review and meta-analysis by Taherifard et al. (2024) found that 75% of patients with SCD revealed abnormal electrocardiogram (ECG) discoveries, comprising prolonged QTc interval and left ventricular hypertrophy, representative of a high prevalence of subclinical and overt cardiac pathology [8]. These abnormalities are repeatedly interconnected to disease severity and haemoglobin levels, underscoring the necessity for comprehensive monitoring [9].

A recently published study by Shdaifat et al. reported that the economic burden caused by SCD in KSA is substantial. It highlighted the need for tailored approaches to address financial challenges and to enhance patient quality of life [10]. Although the national prevalence and economic burden in KSA are becoming clearer, regional heterogeneity demands localized data. The Jazan region is known to have a high prevalence of SCD, with one study indicating that approximately one-fourth of hospital admissions were related to the disease, with VOC and Acute Chest Syndrome (ACS) being the most frequent complications [11]. The Jazan region, with its distinct socioeconomic, predominantly rural demographic profile, and a high 22.6% SCD prevalence among hospital admissions compared with other regions of the Kingdom [11], underscores the critical need for region-specific assessment to guide healthcare policy. Conversely, a comprehensive evaluation of the multifaceted problem of SCD—encompassing its clinical, humanistic, and economic dimensions—specifically in the Jazan population, is lacking.

The present study aims to comprehensively evaluate the impact of SCD on individuals residing in the Jazan region of Saudi Arabia. By simultaneously assessing health challenges, quality of life, and financial burden, our findings will provide crucial evidence to inform regional public health policies, augment resource allocation, and ultimately improve care for this vulnerable patient population.

## Materials and methods

### Ethics

Before the commencement of the study, approval was obtained from the Standing Committee for Scientific Research (HAPO-10-Z-001) at Jazan University, Saudi Arabia, with approval number REC-45/11/1140.

### Study design and setting

A cross-sectional observational study was conducted in the Jazan province of KSA, and the survey was conducted from June 9, 2024 to November 12, 2024. The study was developed as a student project, and the participating students underwent training during their internship to help participants administer the survey digitally and perform data entry.

### Study population

#### Study population and recruitment.

We recruited patients with confirmed sickle cell disease (SCD) attending haematology clinics and hospital pharmacies of public hospitals (Ministry of Health/general and referral hospitals) in Jazan province during the study period. Eligible participants were adults and adolescents aged ≥15 years; participants <18 years completed the survey in the presence of a legally authorized guardian.

As SCD is relatively uncommon and there is no practical population-based sampling frame for all individuals with SCD in the region, we used a clinic-based purposive (consecutive-encounter) sampling approach. Potentially eligible patients were informed about the study during routine care encounters, and those who provided written informed consent were enrolled. We did not maintain a complete screening log of all eligible patients seen across sites (including those not approached), and therefore, a site-level denominator and response rate could not be estimated reliably. Accordingly, findings primarily reflect SCD patients engaged in care at participating facilities and may not be generalisable to individuals with SCD who do not attend these services.

### Sample size calculation

The sample size was calculated using Cochran’s formula for a large population to calculate the required sample size [12]:

n = (zα)^2^ p (1 – p) / d^2^

n = sample size

z = level of confidence according to the standard normal distribution (for a level of confidence of 95%, z = 1.96)

p = prevalence for the study (2.6 percent from the study conducted by Jastaniah et al [6])

d = margin of error (3%)

The estimated sample size was 108 at a 5% absolute precision level and a 5% standard normal deviation.

### Data collection tool

The data were collected using a self-administered validated questionnaire prepared after a thorough literature search [13–16]. The structured questionnaire had several sections to gather information from the eligible respondents. The first part of the questionnaire gathered information related to sociodemographic characteristics. The second part consisted of 8 open-ended questions to assess the direct and indirect expenditure burden experienced by the respondents with SCD. The third section consisted of the Arabic version of questions regarding the impact on mental health (using the Kessler Psychological Distress Scale 6 (K6)) [13], pain (using the Pain Catastrophizing Scale-Hopelessness) [14], physical health, and social life (using the WHOQOL-100) [15]. All the items in the 3^rd^ section were on a 5-point scale. The final section of the questionnaire consisted of questions regarding the medical and surgical treatments the respondent had received as part of the SCD management, as well as their perceptions regarding the health care providers and services they had received.

The survey details were provided verbally before the survey began, and an electronic informed consent was obtained before proceeding with the digital survey. Respondents below the age of 18 years were surveyed in the presence and with the consent of either one of their parents. No personal identifying information was collected during the survey, and the respondents were assured of the confidentiality of their information.

### Measures

1. **Demographics:** The variables consisted of sex (male, female), age in years, Nationality (Saudi, non-Saudi), weight in kg, height in cm, number of family members, monthly family income, educational level (high school or less, university degree or higher), employment status (student, employed, unemployed), marital status (single, married), area of residence (city, village, mountain area), and diagnosis of chronic physical or mental conditions (yes, no). The chronic conditions included hypertension, heart disease, diabetes, dyslipidemia, arthritis, gastrointestinal diseases, neurological or psychiatric disorders, and others.
2. **Financial burden:** The survey consisted of open-ended questions that assessed both the direct medical costs (out-of-pocket (OOP) expenses for hospitalizations, consultations, laboratory investigations, medications, home care services, etc) and the direct non-medical costs (OOP expenses for transportation, food, accommodation, etc) that arose due to sickle cell disease. High OOP spending burden was defined as spending 10% or more of family income on SCD-related healthcare [17]. The costs were collected in Saudi Arabian Riyals (SAR) and were additionally reported in U.S. Dollars (USD) using the fixed exchange rate of 1 USD = 3.75 SAR, which has been in effect since 1986 [18].
3. **Missed work/school days:** The patient’s missed work/school days due to hospital visits, pain, and other distressing symptoms, as well as the missed work/school days of caregivers, were collected using open-ended questions.
4. **Psychological Distress:** Mental health burden was assessed using the Kessler-6 (K6) scale, which consisted of six items evaluating the frequency of experiencing symptoms such as feeling nervous, hopeless, restless, depressed, worthless, and that everything was an effort. Responses were recorded on a 5-point scale ranging from “always” to “never” [13]. The total K6 score ranges from 0 to 24, with scores of 13 or higher indicating serious psychological distress (SPD)—a validated indicator of the probability of mental illness [19–21]. Based on this threshold, participants were classified as having SPD (K6 ≥ 13) or not (K6 < 13).
5. **Physical Health:** The overall physical health was measured using six items on a 5-point scale ranging from ‘Strongly Disagree’ to ‘Strongly Agree’ assessing the dissatisfaction with physical health, inability to get sufficient sleep due to SCD symptoms, failure to perform daily activities, dependence on medications or devices for daily tasks, inability to work consistently and inability to find employment due to physical health problems [15].
6. **Pain:** The burden of pain was assessed using six items derived from the original Pain Catastrophizing Scale [14]. Items were rated on a 5-point Likert scale from “Strongly Disagree” to “Strongly Agree.” The items included questions regarding whether there was constant worry if the pain would ever end, whether the person could not continue with the experience of the pain, whether the pain could not be improved, if the pain was exhausting, if the pain could no longer be borne, and if the intensity of the pain could not be reduced. As only a subset of the original items was used, exploratory factor analysis (EFA) was conducted to examine the underlying structure of the adapted scale in our study population.
7. **Social Life:** The impact on social life was measured using five items assessing various aspects of social life on a 5-point scale ranging from ‘Strongly Disagree’ to ‘Strongly Agree.’ The survey evaluated whether individuals were satisfied with their personal relationships, whether they received sufficient help and support from those around, whether the ability to help others readily and willingly was unaffected by SCD, whether SCD impacts social relationships with colleagues, and whether SCD affects interaction with the public in public places [15].
8. **Interaction with healthcare providers:** This section was measured using 10 items that were partially adapted from the Sickle Cell World Assessment Survey (SWAY) by Osunkwo et al. (2021) [16], which were refined to align with the objectives and context of this study. Responses were recorded on a 5-point Likert scale ranging from “Strongly Disagree” to “Strongly Agree.” The survey evaluated the respondents’ experience regarding interaction with healthcare providers, including the perceived interest of the healthcare provider in helping, symptom inquiry during each visit, physician’s understanding of the impact of the disease on the respondents’ lives, patient’s comfort in discussing symptoms with the physician, trust in treatment and disease monitoring, if the physician discusses potential side effects of the medications and new treatment options, if the physician shared treatment goals with the respondent, whether optimal management of SCD was not offered to the respondent, and patient avoidance of physician visits due to fear.
9. **Healthcare utilization:** Healthcare utilization was measured using multiple-choice questions with multiple answer options regarding medications and surgeries commonly utilized in SCD management.

### Statistical analysis

Data were analyzed using STATA version 18. All figures were produced using RStudio. Descriptive statistics were generated to summarize participant characteristics and were recorded as counts, percentages, means, and standard deviations. Chi-square tests were conducted to examine differences in demographic categorical variables between individuals with no high spending burden versus those with a high spending burden, as well as between those with and without SPD. Similarly, two-tailed t-tests were performed to assess differences in continuous demographic variables across both comparisons. For each scale used in this study, an EFA was conducted using principal component extraction. The suitability of data for factor analysis was assessed using Bartlett’s test and the Kaiser-Meyer-Olkin (KMO) test to check sampling adequacy [22]. Velicer’s test established the number of components [23]. Confirmatory factor analysis (CFA) was conducted using structural equation modeling (SEM) in STATA 18 with maximum likelihood with the missing values estimation method. Model fit was assessed using multiple indices: the chi-square test, root mean square error of approximation (RMSEA) with its 90% confidence interval, comparative fit index (CFI), Tucker–Lewis index (TLI), standardized root mean square residual (SRMR), Akaike information criterion (AIC), and Bayesian information criterion (BIC). Acceptable model fit was defined by RMSEA ≤ 0.08, CFI and TLI ≥ 0.95, and SRMR ≤ 0.08. Model comparisons were evaluated using the likelihood ratio test (Δχ²) to determine whether the two-factor model significantly improved model fit over the one-factor structure for Kessler-6 scale. Given the modest sample size (N = 110), the psychometric results should be interpreted with caution, as both EFA and CFA were conducted on the same sample. These analyses were intended to provide preliminary insight into scale properties in this population rather than to establish definitive validation.

## Results

### Demographics

The study enrolled all 110 eligible individuals who consented to participate in the study during the defined study period. The mean age of the participants was 28.43 (SD ± 9.66) years. Most respondents were male (55.5%), and only 4.5% were non-Saudi. Regarding their educational qualifications, 46.4% had completed high school, while 53.6% held a university degree or higher. The employed and unemployed were in equal proportion (36.4%), while 27.3% were students. Among the respondents, 62.7% were single, 10.9% lived in mountain regions, while 47.3% and 41.8% resided in cities and villages, respectively. Only 26.4% of respondents had chronic health conditions, and the mean monthly family income was 14,597.92 SAR (3,892.78 USD) (Table 1).

**Table 1:** Demographic, socioeconomic, clinical characteristics, and healthcare utilization among individuals with sickle cell disease.

Variables	Summary
N	110
Age	28.427 (9.657)
Sex	
Male	61 (55.5%)
Female	49 (44.5%)
Nationality	
Saudi	105 (95.5%)
Non-Saudi	5 (4.5%)
Family Income (monthly)	14,597.92 ± 20,068.64 SAR (3,892.78 ± 5,351.64 USD)
Education	
High school	51 (46.4%)
University degree or higher	59 (53.6%)
Job Status	
Student	30 (27.3%)
Employed	40 (36.4%)
Unemployed	40 (36.4%)
Marital status	
Single	69 (62.7%)
Married	41 (37.3%)
Area	
City	52 (47.3%)
Village	46 (41.8%)
Mountain area	12 (10.9%)
Other Chronic conditions	
Yes	29 (26.4%)
No	81 (73.6%)
OOP Expenditures (Medications)	379.48 ± 812.51 SAR (101.2 ± 216.67 USD)
OOP Expenditures (Home care)	446.21 ± 3,183.98 SAR (118.99 ± 849.06 USD)
OOP Expenditures (Hospital)	561.46 ± 682.83 SAR (149.72 ± 182.09 USD)]
OOP Expenditures (Transportation)	828.51 ± 925.84 SAR (220.94 ± 246.89 USD)]
Missed Work/School Days due to hospital visits	4.173 (5.070)
Missed Work/School Days – Caregiver	3.936 (3.585)
Missed Work/School Days due to pain	4.718 (4.046)
Serious Psychological Distress	
No-Distress	45 (40.9%)
Distress	65 (59.1%)
High OOP Spending burden	
No	48 (44.9%)
Yes	59 (55.1%)
Hydroxyurea	
No	20 (18.2%)
Yes	90 (81.8%)
L-glutamine	
No	103 (93.6%)
Yes	7 (6.4%)
Crizanlizumab	
No	103 (93.6%)
Yes	7 (6.4%)
Opioid Analgesics	
No	58 (52.7%)
Yes	52 (47.3%)
Other analgesics	
No	47 (42.7%)
Yes	63 (57.3%)
Other anti-inflammatory drugs	
No	93 (84.5%)
Yes	17 (15.5%)
Iron chelators	
No	99 (90.0%)
Yes	11 (10.0%)
Folic	
No	31 (28.2%)
Yes	79 (71.8%)
Vitamin D	
No	49 (44.5%)
Yes	61 (55.5%)
No Medications	
No	100 (90.9%)
Yes	10 (9.1%)

### Financial burden and missed work/school days

The burden of high OOP spending was seen among 55.1% of the respondents. The highest expenditure was on transportation to receive medical care (828.51 ± 925.84 SAR/220.94 ± 246.89 USD) per month, followed by hospitalization, lab tests & treatments. (Table 1). The self-reported approximate missed days of respondents in a month were 4.72 (SD ± 4.05) days from work/school due to pain and lost 4.17 ± 5.07 days due to hospital visits, while caregivers (family/relatives) lost 3.94 (SD ± 3.59) days from work/school (Table 1).

The OOP spending among participants was evaluated by comparing those who experienced a high burden of spending with those who did not. There was no statistical difference between groups regarding age, sex, education level, employment status, or area of residence. In the high spending burden group, the average monthly family income and physical health score were significantly lower (*p* = 0.014 and *p* < 0.001, respectively), and the pain score tended to be lower (*p* = 0.072) without reaching statistical significance (Table 2).

**Table 2:** Comparison of demographic, socioeconomic, and health-related characteristics between individuals with and without high out-of-pocket spending burden for sickle cell disease.

OOP Spending Burden			
Variables	No High spending burden	High OOP Spending Burden	Test
N	48 (44.9%)	59 (55.1%)	
Age	29.500 (10.717)	27.864 (8.825)	0.389^*^
Sex			
Male	26 (54.2%)	34 (57.6%)	0.720^$^
Female	22 (45.8%)	25 (42.4%)	
Education			
≤ High School	22 (45.8%)	27 (45.8%)	0.994^$^
College/above	26 (54.2%)	32 (54.2%)	
Marital status			
Single	28 (58.3%)	39 (66.1%)	0.409^$^
Married	20 (41.7%)	20 (33.9%)	
Job Status			
Student	14 (29.2%)	15 (25.4%)	0.881^$^
Employed	18 (37.5%)	22 (37.3%)	
Unemployed	16 (33.3%)	22 (37.3%)	
Area			
City	24 (50.0%)	28 (47.5%)	0.957^$^
Village	19 (39.6%)	25 (42.4%)	
Mountains	5 (10.4%)	6 (10.2%)	
Family Income (Monthly)	20,304.33 ± 28,480.97 SAR (5,414.49 ± 7,594.93USD)	10,697.68 ± 6,791.43 SAR (2,852.71 ± 1811.05USD)	0.014^*^
Physical Score	17.917 (6.620)	13.695 (5.946)	<0.001^*^
Pain Score	17.833 (7.419)	15.288 (7.044)	0.072^*^
Social Score	8.250 (3.884)	9.373 (3.850)	0.138^*^

* Student’s t test. ^$^ Pearson’s 𝜒2 test.

### Psychometric overview (Exploratory factor analyses)

EFA indicated adequate sampling for all domains and suggested unidimensional structures with strong internal consistency. CFA results supported the two-factor structure of the K6 and provided a reasonable model fit for the physical domain. The social domain showed weaker model fit, suggesting the scale may not fully capture a coherent construct in this sample.

Complete model statistics and factor loadings are reported in Appendix 1.

### Psychological distress

SPD was predominant in 59.1% of the respondents (Table 1). SPD was significantly associated with older age (*p* = 0.008), being married (50.8% vs. 17.8%, *p* = 0.001), and employed (49.2% vs. 17.2%, *p* = 0.003). Social scores were significantly lower among participants with SPD (*p* = 0.006), whereas physical health scores and pain scores were significantly higher in the SPD group (*p* < 0.001). The other variables studied (sex, education, area of residence, monthly family income, chronic conditions) did not differ between groups (Table 3).

**Table 3:** Comparison of demographic, socioeconomic, and health-related characteristics between individuals with and without serious psychological distress.

Serious Psychological Distress			
Variables	No	Yes	Test
N (%)	45 (40.9%)	65 (59.1%)	
Age	25.533 (7.197)	30.431 (10.642)	0.008*
Sex			
Male	26 (57.8%)	35 (53.8%)	0.863^$^
Female	19 (42.2%)	30 (46.2%)	
Education			
≤ High School	24 (53.3%)	27 (41.5%)	0.223^$^
College/above	21 (46.7%)	38 (58.5%)	
Marital status			
Single	37 (82.2%)	32 (49.2%)	0.001^$^
Married	8 (17.8%)	33 (50.8%)	
Job Status			
Student	16 (35.6%)	14 (21.5%)	0.003^$^
Employed	8 (17.8%)	32 (49.2%)	
Unemployed	21 (46.7%)	19 (29.2%)	
Area			
City	19 (42.2%)	33 (50.8%)	0.623^$^
Village	20 (44.4%)	26 (40.0%)	
Mountains	6 (13.3%)	6 (9.2%)	
Chronic Conditions			
Yes	16 (35.6%)	13 (20.0%)	0.069^$^
No	29 (64.4%)	52 (80.0%)	
Family Income	16,931.78 ± 30,282.54 SAR (4515.14 ± 8075.34 USD)	12,982.17 ± 6,999.517 SAR (3461.91 ± 1866.54 USD)	0.312^*^
Social score	10.067 (3.532)	8.000 (3.913)	0.006*
Physical score	12.622 (5.245)	17.754 (6.605)	<0.001^*^
Pain score	12.044 (5.460)	19.892 (6.778)	<0.001^*^

* Student’s t test. ^$^ Pearson’s 𝜒2 test.

### Physical health

**Fig 1:**
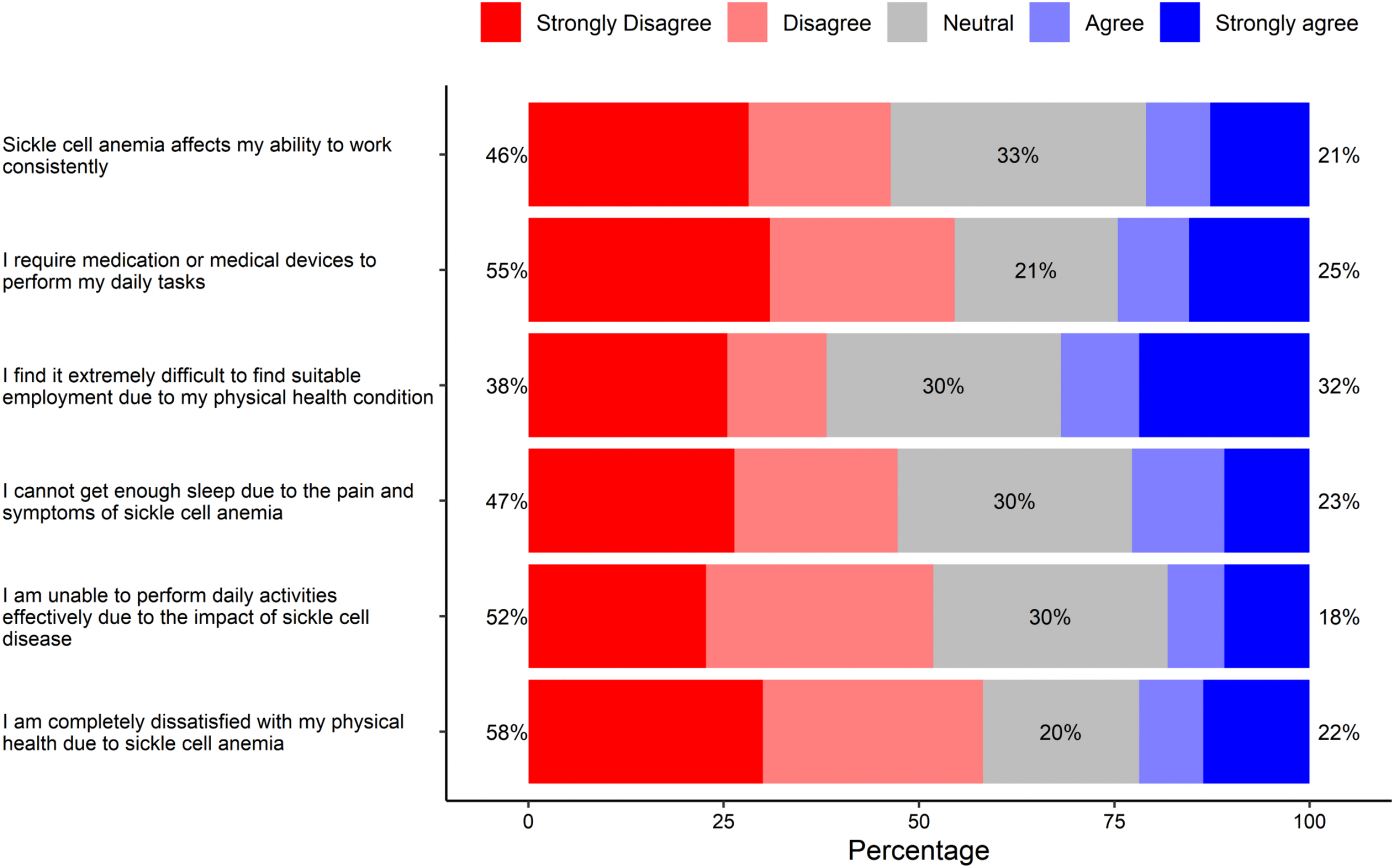
The impact of sickle cell disease on physical health.

The impact of SCD on physical health is presented in Fig 1. Approximately 46% of respondents disagreed that SCD interfered with their occupational activities, and more than half (55%) of them indicated that they did not need medication or medical devices to perform their daily functions. About one-third (32%) of respondents found it challenging to get employment due to their physical health limitations. SCD-related symptoms did not cause sleep disturbances for nearly (47%) of the respondents. A majority (52%) reported no limitations to perform daily activities due to SCD, and 58% disagreed that they were dissatisfied with their physical health due to SCD.

### Pain

**Fig 2:**
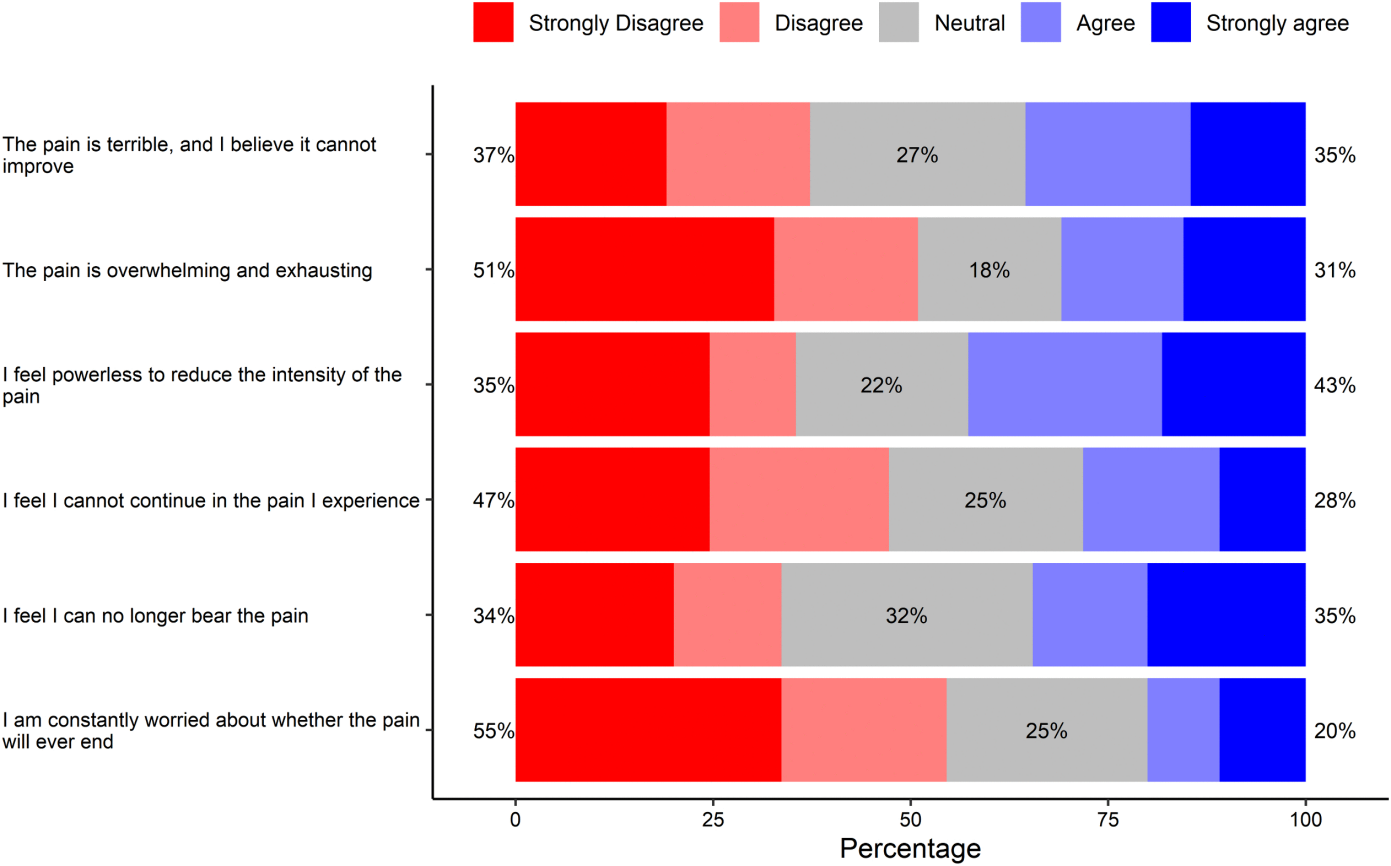
The impact of pain in sickle cell disease patients.

The perception about pain and the potential for it not to improve were mixed and less pronounced, with over one-third of participants each agreeing and disagreeing. The pain was not overwhelming or exhaustive for 51% of respondents. Perceptions of pain tolerance were divided, with approximately one-third of respondents agreeing and one-third disagreeing that they can bear the pain. Forty-three percent of respondents felt powerless to reduce symptoms of pain, but 47% of respondents felt they could continue with the pain they experienced (Fig 2).

### Social life

**Fig 3:**
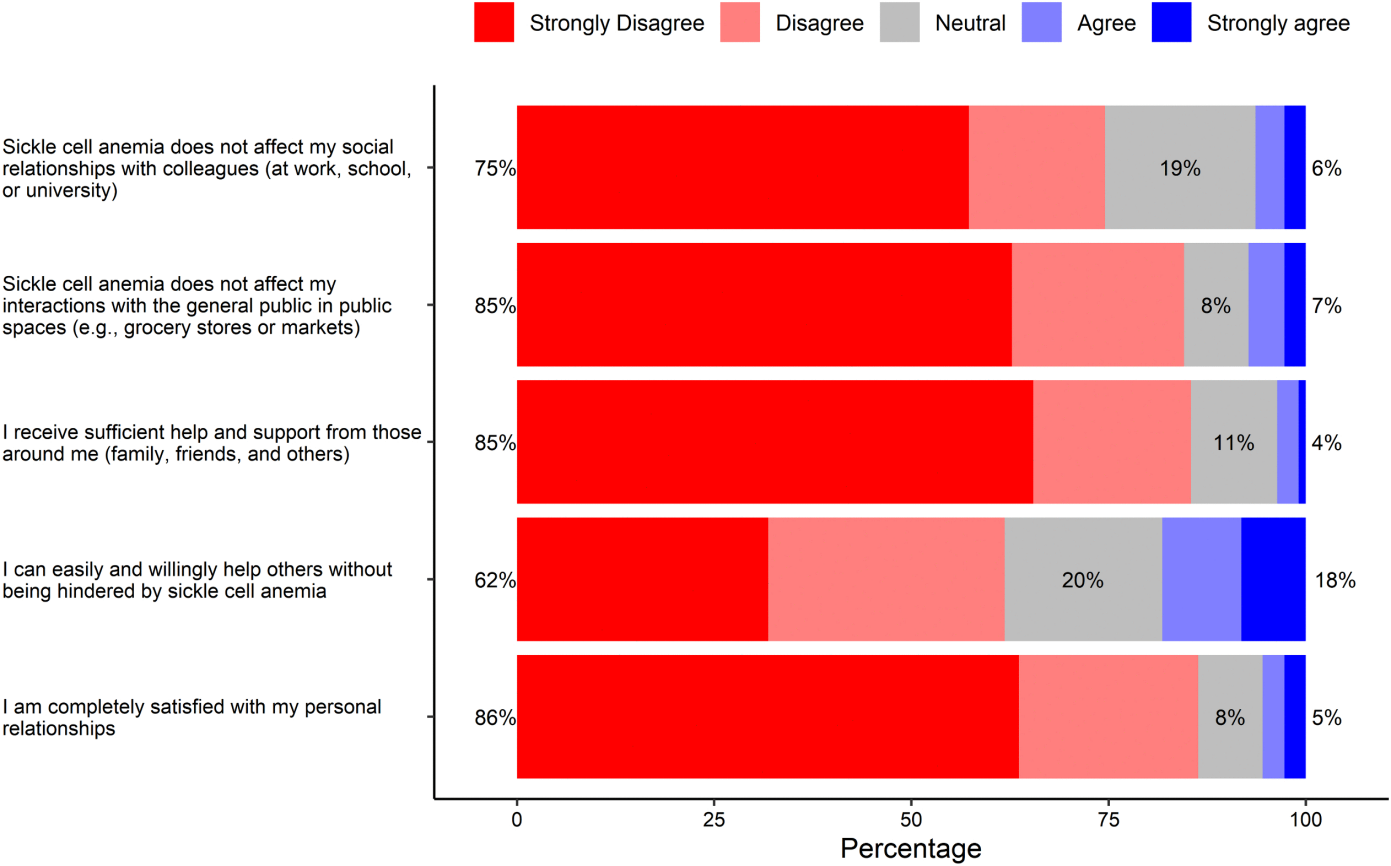
The impact of sickle cell disease on interpersonal relationships and support systems.

Most participants (85%) believed their social interaction with the public was affected, while a similar number of respondents (86%) felt not completely satisfied with their personal relationships. Approximately three-fourths of the respondents implied that their condition affected their social interactions with colleagues at work, school, or university, and the majority (85%) perceived that the support from family and friends was not adequate. About two-thirds of respondents (62%) felt SCD affected their willingness to help others (Fig 3).

### Healthcare utilization

Hydroxyurea was the most used medication (81.8%). L-glutamine and crizanlizumab were each used by 6.4% of the respondents. Opioid analgesics were used by 47.3% of respondents, while other analgesics were used by 57.3%. Additionally, 15.5% used anti-inflammatory agents other than non-steroidal anti-inflammatory drugs. Iron chelators were used by 10%, vitamin D by 55.5%, and folic acid by 71.8% of the respondents (Table 1).

Overall, 47.3% of patients underwent at least one surgical procedure, with splenectomy and cholecystectomy performed in 25.5% and 19.1% of patients, respectively. Tonsillectomy was done in 8.2% of cases, while orthopedic, ophthalmic, and renal surgeries were less frequent. Liver-related surgeries were not reported (Table 4).

**Table 4:** Surgical procedures among individuals with sickle cell disease (SCD).

Variables	N (%)
N	110
Spleen Surgery	
No	82 (74.5%)
Yes	28 (25.5%)
Tonsilitis Surgery	
No	101 (91.8%)
Yes	9 (8.2%)
Gallbladder Surgery	
No	89 (80.9%)
Yes	21 (19.1%)
Orthopedic surgery	
No	107 (97.3%)
Yes	3 (2.7%)
Ophthalmic surgery	
No	108 (98.2%)
Yes	2 (1.8%)
Liver Surgery	
No	110 (100.0%)
Renal surgery	
No	107 (97.3%)
Yes	3 (2.7%)
Other surgeries	
No	106 (96.4%)
Yes	4 (3.6%)
No Surgeries	
No	58 (52.7%)
Yes	52 (47.3%)

### Interaction with Healthcare providers

**Fig 4:**
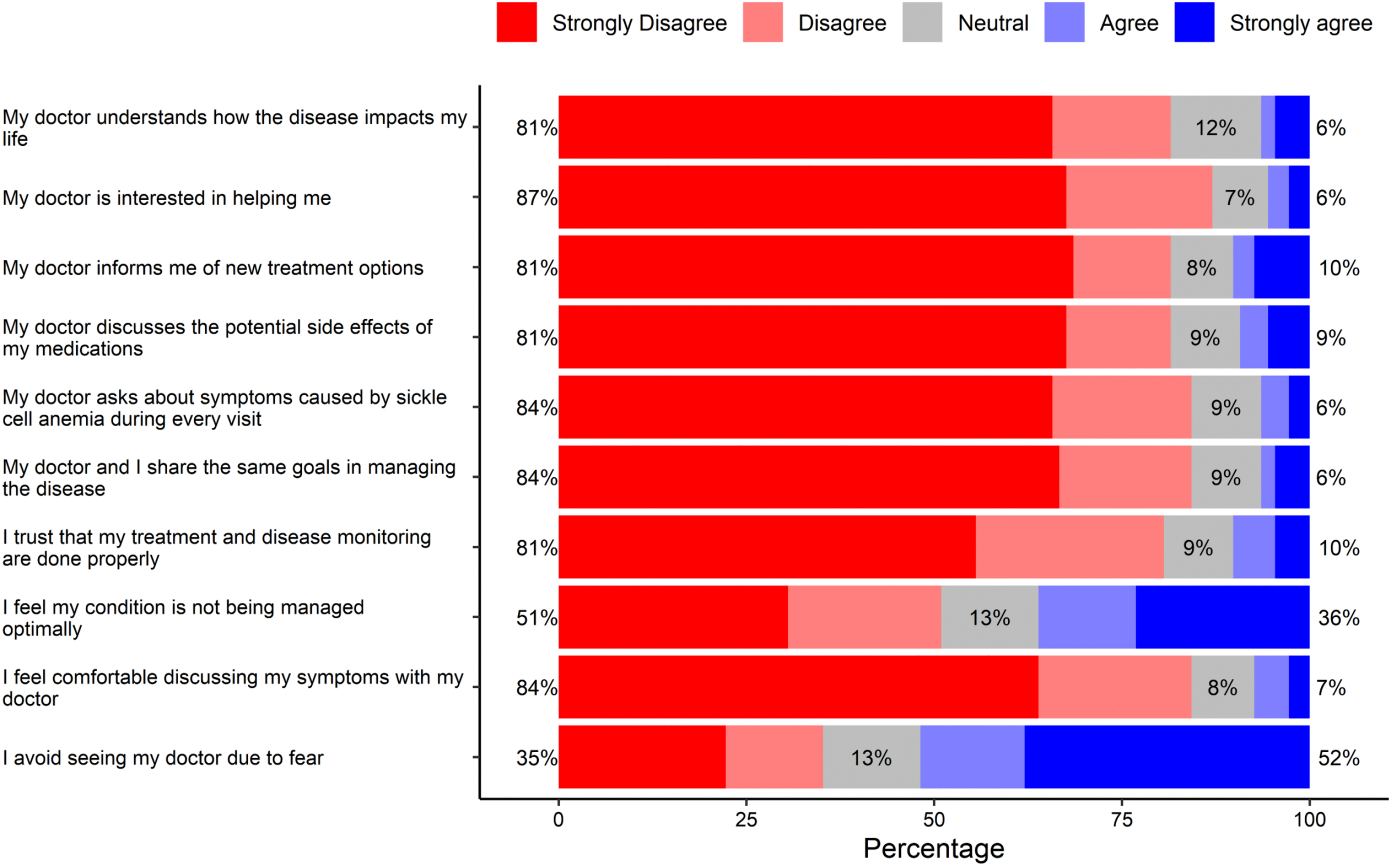
The perception SCD patients had about their physician.

Fig 4 highlights the perception SCD patients had about their physician. Eighty-one percent felt that their physician did not understand the impact of the disease on their life, while a higher percentage (87%) felt that their doctor lacked interest in helping them. Eighty-one percent of the patients said that their physician did not discuss treatment goals with them, with the same proportion admitting that medication side effects were not adequately discussed. The majority (84%) felt that their physician did not address symptoms caused by SCD at every visit and did not share the same goals in disease management, while 6% agreed with these statements. Similarly, 84% disagreed that they were comfortable discussing symptoms with their physician. Trust in the quality of care was low, with 81% strongly disagreeing that their disease is appropriately monitored. Although 36% agreed that their condition is not managed optimally, 51% disagreed. Most respondents were uncomfortable discussing their symptoms with their physician, and 52% avoided physician visits due to fear.

## Discussion

The present study identified socioeconomic, healthcare, and psychological distress experienced by individuals with SCD in Saudi Arabia.

The respondents comprised young adults in their late twenties, with a slightly higher male representation, consistent with other studies in Saudi Arabia reporting a similar age range of 28–29 years [24,25]. Understanding the age distribution is essential, as it represents a stage in life when careers, marriage, and social roles are consolidated; yet, the burden of recurrent symptoms and health issues can disrupt personal, professional, and social life. The minimal representation of non-Saudis in our sample indicates that SCD is an indigenous disorder in the Saudi population and has been consistently reported as a common genetic disorder in Saudi Arabia [26,27].

Despite many participants holding university degrees, their employment outcomes remained concerning, with only a little over one-third employed. Some participants reported health-related limitations that could hinder sustained employment, although many did not endorse work interference. These findings align with the results of Shdaifat et al., who reported difficulty securing and maintaining employment among individuals affected by this chronic health condition [10]. Fatigue, VOCs, and musculoskeletal complications impair work capabilities. Similarly, a U.S.-based study reported that one in five patients with SCD experience recurrent hospital visits, pain, psychological distress, and overall disability, which served as barriers to employment [28]. Our findings also showed that employment itself may have been associated with distress among respondents, possibly due to the difficulty in balancing job demands with disease management.

The financial hardships faced by patients and their families were evident, with over half of the respondents experiencing high OOP expenses. Transportation alone averaged 828.51 SAR (220.94 USD), restricting access to specialized healthcare services. The findings align with a U.S. study by Smeltzer et al., which reported reduced hospitalization among those living far away from the health facility, despite reimbursement of transportation costs [29]. Such expenses, along with hospitalization, medications, and home care costs, induce further financial strain. Ohaeri and Shokunbi have emphasized that the financial costs surpass the direct costs of managing the disease itself [30]. Interestingly, single respondents spent more on managing their health than their married counterparts, though the difference was not statistically significant, likely due to a lack of shared financial support [31]. Men also reported higher OOP expenditure, consistent with studies showing that males with SCD often experience painful crises attributable to their physical activity and participation in sports [32,33]. In case of low-income families, the burden was higher, which, as evidenced earlier, could eventually lead to delayed care or abstaining from treatment, resulting in higher morbidity in SCD patients [34].

Although the Saudi Ministry of Health covers direct costs through the national healthcare model, the high OOP expenses reflect possible gaps in healthcare coverage [25], particularly in supportive care, transport, and access to novel therapies. Implementing inclusive insurance schemes could be one effective strategy to alleviate the financial burden. Such reforms align with Saudi Vision 2030, which aims to reduce the financial burden and enhance healthcare accessibility through its transformative goals [35]. Extension of the health financing model beyond hospital-based care could significantly improve the quality of life and reduce the economic burden on families in the long run.

Approximately 60% of the respondents reported SPD. It may be related to pain, recurrent hospitalizations, and the unpredictable course of the disease [36–39]. SPD was significantly associated with age and marital status in our sample, with 50.8% of those with SPD being married. Among individuals without SPD, the majority were single individuals (82.2%), while only 17.8% of the married individuals reported no SPD. This finding contrasts with a study conducted in Saudi Arabia that reported higher distress levels among single individuals with SCD [40], while a study from Jazan further indicated that divorced or widowed patients experience higher levels of distress compared to single or married groups [41]. Taken together, it shows that the relationship between marital status and psychological distress is not straightforward but is shaped by several social and cultural factors. In our data, distressed respondents also exhibited lower social functioning scores, pointing to the influence of social isolation, limited support systems, or changing family dynamics [36]. Relationship challenges were evident, with only 4% of respondents feeling supported by their family and friends. This aligns with the findings of a Jamaican study, which identified a lack of family support as a contributor to psychological distress [42]. These patterns show that marital status alone cannot explain psychological distress, but the quality of social support and economic pressures contribute to the burden of distress in patients with SCD. The results suggest the need for psychosocial services to strengthen social inclusion, which might help in alleviating distress.

Health issues can lead to absenteeism from work, which can result in financial instability. Self-reported approximate missed work or school days due to painful episodes and hospital visits indicate functional impairments faced by SCD-affected individuals. In our study, acute complications such as splenic procedures and cholecystectomy were seen more frequently compared with other health issues. These findings align with previous research conducted in Saudi Arabia and England, which identified gallstones as a common complication associated with SCD [31,34,43]. Schwartz et al. have reported absenteeism as a significant problem among adolescents afflicted with SCD [44]. Caregivers are also affected, as they miss an average of 3.94 workdays, indicating the socioeconomic impact of SCD on families.

Nociceptive pain remains a significant problem in SCD, with some reporting lower tolerance, while others experience exhaustion. Respondents had higher pain scores, validating the relationship between pain and psychological health in SCD [45]. Pain affects emotional well-being and could be a predictor of psychological distress [36]. Addressing this requires effective pain management strategies, including pharmacological and non-pharmacological interventions, to improve psychological well-being [46].

Chronic pain detrimentally impacts daily functioning, employment, and psychological well-being. Evensen et al. have pointed out that two-thirds of the respondents had severe pain, which disrupted their daily tasks [47], consistent with our findings. The analgesic use pattern further offers insight into the disease burden. More than half reported the use of non-opioid analgesics, while nearly half required opioids, reflecting the burden of pain in SCD. The trend is consistent with the global practice, where opioids remain essential for managing VOCs. Unfortunately, patients presenting prescriptions for opioid analgesics are often stigmatized and misclassified as drug-seekers, contributing to delays in treatment and pain control [48]. The delayed treatment could have been perceived as a lack of physician empathy and effective communication by our sample.

Hydroxyurea utilization in our sample (81.8%) is consistent with global recommendations and similar studies in Saudi Arabia [49,50], reflecting its established role as the standard of care for reducing VOC episodes [51–57]. In contrast, newer agents like L-glutamine and crizanlizumab showed minimal use, likely due to formulary restrictions and market withdrawals [58]

Iron chelators, on the other hand, were prescribed to a smaller proportion of patients. Chelation is indicated only for patients facing transfusion-related iron overload, and in our sample, only a few patients needed intermittent blood transfusions to manage acute complications.

A substantial proportion of respondents reported challenges in interactions with their physicians, including discomfort in discussing symptoms. Approximately half indicated avoiding physician visits due to fear, and fewer than half perceived that they received optimal care. Evenson et al. reported that a high proportion of the respondents had negative experiences in emergency department settings, which was consistent with our findings [47]. Similarly, Carvalho et al. reported concerns regarding pain assessment and delay in care provided to patients with SCD [59]. A study in Saudi Arabia has also reported poor service and communication from healthcare professionals [60]. Global experiences suggest pathways for improvement. For example, the UK National Health Service (NHS) has introduced expert clinics that provide specialized care. Such models reduce waiting times in emergency departments, ensuring compassionate and timely management. Adapting similar strategies in local healthcare systems could improve outcomes and improve the quality of life [61].

The study highlights the urgent need for a large-scale regional evaluation to confirm the findings and to re-evaluate the health care services provided to SCD patients. Based on this evidence, policy reforms should be aimed at reducing the economic and psychological burden of SCD. Measures like modifying insurance coverage to include transportation in addition to medications or providing free home health care services could mitigate the OOP expenses. Multidisciplinary approaches to pain management, along with mental health support in routine SCD care, can immensely help in addressing the high prevalence of SPD. Physician training also needs to be emphasized.

### Limitations

This study has several limitations. Firstly, the cross-sectional, clinic-based purposive sampling approach may introduce selection bias and limit the external validity. Participants were recruited from haematology clinics and hospital pharmacies at participating facilities; therefore, findings are most generalisable to SCD patients who are actively engaged in care, and may not reflect individuals with SCD who do not attend these services or who seek care elsewhere. In addition, because a comprehensive screening log of all eligible patients seen across sites (including those not approached) was not systematically maintained, a site-level denominator, response rate, and reasons for non-participation could not be estimated reliably.

Secondly, the sample size was modest, and some subgroups (e.g., non-Saudi respondents and adolescents <18 years) were underrepresented, limiting the precision of subgroup comparisons and precluding reliable age-stratified multivariable analyses.

Additionally, the exploratory and confirmatory factor analyses should be interpreted with caution due to the limited sample size (N = 110). The sample may be insufficient for stable factor analysis across the number of items examined, and therefore, the identified factor structures should be considered exploratory rather than definitive.

Finally, the outcomes were self-reported, which may be subject to recall and reporting bias, particularly for OOP expenses and healthcare utilization. Fourth, the analysis focused on direct OOP costs and did not capture indirect costs (e.g., productivity loss, caregiver time), which may underestimate the overall economic burden. Finally, measures of patient–physician interactions were based on patient perceptions and may reflect subjective experiences.

## Conclusion

This study highlights the burden that SCD imposes on individuals and their families, impacting their economic, psychological, and social well-being. Pain and functional impairments can lead to frequent hospitalizations. These, in turn, can lead to absenteeism from work or school, further adding to the financial strain. Difficulties in finding employment, poor social interaction, and a perceived lack of family support can be contributing factors to SPD. Further, the high OOP expenses among families with limited resources can add to the burden. Despite free healthcare coverage for Saudi citizens, OOP expenses for transportation, specialized care, and home-based services persist. Despite the availability of free public healthcare services, these findings suggest potential opportunities to enhance insurance coverage and reduce OOP healthcare-related expenses for individuals with chronic disorders. Strengthening the empathetic engagement of the healthcare team also represents a key opportunity to further support the complex needs of the SCD population.

## Supporting information

S1 File Appendix 1 (model statistics and factor loadings). (DOCX)

S2 File Survey in Arabic. (DOCX)

S3 File Survey in English. (DOCX)
