# Supplementary material for: Clinical, humanistic, and economic burden of sickle cell disease in The Jazan Region, Saudi Arabia
Source: PLoS One. 2026 May 14;21(5):e0348759. doi: 10.1371/journal.pone.0348759 (PMC13175482; doi:10.1371/journal.pone.0348759)
Supplement: S1 File — (DOCX) [file pone.0348759.s001.docx]

Appendix 1: EFA and CFA Results

Kessler-6

| Item  During the past 30 days, about how often did you feel | Single Factor Loadings | Cronbach’s Alpha |
| --- | --- | --- |
| nervous? | 0.7102 | 0.91 |
| hopeless? | 0.8072 |  |
| restless or fidgety? | 0.8506 |  |
| so depressed that nothing could cheer you up? | 0.8505 |  |
| that everything was an effort? | 0.7649 |  |
| worthless? | 0.7425 |  |
| Kaiser–Meyer–Olkin (KMO) test for sampling adequacy | 0. 871 | |
| Bartlett’s Test of Sphericity | | |
| Approximate Chi square | 415.793 | |
| DF | 15 | |
| Significance | <0.001 | |

Physical Health

| Item | Single Factor Loadings | Cronbach’s Alpha |
| --- | --- | --- |
| I am completely dissatisfied with my physical health due to sickle cell anemia. | 0.7194 | 0.90 |
| I cannot get enough sleep due to the pain and symptoms of sickle cell anemia. | 0.8438 |  |
| I am unable to perform daily activities effectively due to the impact of sickle cell disease. | 0.9096 |  |
| I require medication or medical devices to perform my daily tasks. | 0.6646 |  |
| Sickle cell anemia affects my ability to work consistently. | 0.7838 |  |
| I find it extremely difficult to find suitable employment due to my physical health condition. | 0.7179 |  |
| Kaiser–Meyer–Olkin (KMO) test for sampling adequacy | 0.866 | |
| Bartlett’s Test of Sphericity | | |
| Approximate Chi square | 400.940 | |
| DF | 15 | |
| Significance | <0.001 | |

Pain

| Item | Single Factor Loadings | Cronbach’s Alpha |
| --- | --- | --- |
| I am constantly worried about whether the pain will ever end. | 0.8512 | 0.95 |
| I feel I cannot continue in the pain I experience. | 0.9289 |  |
| The pain is terrible, and I believe it cannot improve. | 0.9032 |  |
| The pain is overwhelming and exhausting. | 0.9091 |  |
| I feel I can no longer bear it. | 0.8988 |  |
| I feel powerless to reduce the intensity of the pain. | 0.7234 |  |
| Kaiser–Meyer–Olkin (KMO) test for sampling adequacy | 0.889 | |
| Bartlett’s Test of Sphericity | | |
| Approximate Chi square | 671.601 | |
| DF | 15 | |
| Significance | <0.001 | |

Social life

| Item | Single Factor Loadings | Cronbach’s Alpha |
| --- | --- | --- |
| I am completely satisfied with my personal relationships. | 0.6659 | 0.82 |
| I receive sufficient help and support from those around me (family, friends, and others). | 0.7113 |  |
| I can easily and willingly help others without being hindered by sickle cell anemia. | 0.4609 |  |
| Sickle cell anemia does not affect my social relationships with colleagues (at work, school, or university). | 0.7965 |  |
| Sickle cell anemia does not affect my interactions with the general public in public spaces (e.g., grocery stores or markets). | 0.8277 |  |
| Kaiser–Meyer–Olkin (KMO) test for sampling adequacy | 0.744 | |
| Bartlett’s Test of Sphericity | | |
| Approximate Chi square | 228.267 | |
| DF | 10 | |
| Significance | <0.001 | |

CFA Model Fit Indices

| Scale | χ²(df), p | RMSEA (90% CI) | CFI | TLI | SRMR | AIC | BIC |
| --- | --- | --- | --- | --- | --- | --- | --- |
| K6 (1-factor) | 248.01 (15), p<0.001 | 0.378 (0.337–0.420) | 0.537 | 0.351 | 0.228 | 2072.8 | 2121.4 |
| K6 (2-factor) | 14.39 (8), p=0.072 | 0.086 (0.000–0.156) | 0.985 | 0.971 | 0.031 | 1857.5 | 1908.8 |
| Physical | 21.91 (9), p=0.009 | 0.115 (0.054–0.177) | 0.968 | 0.946 | 0.042 | 1899.8 | 1948.4 |
| Social | 33.41 (5), p<0.001 | 0.228 (0.159–0.305) | 0.874 | 0.748 | 0.079 | 1402.0 | 1442.5 |
