# Supplementary material for: Clinical, humanistic, and economic burden of sickle cell disease in The Jazan Region, Saudi Arabia
Source: PLoS One. 2026 May 14;21(5):e0348759. doi: 10.1371/journal.pone.0348759 (PMC13175482; doi:10.1371/journal.pone.0348759)
Supplement: S2 File — (DOCX) [file pone.0348759.s002.docx]

**هل تم تشخيصك من قبل طبيب مختص بمرض الأنيميا المنجلية؟**

 - نعم

- لا

**المعلومات الشخصية:**

**الجنس:**

- ذكر

- أنثى

**الجنسية:**

- سعودي/ة

- غير سعودي/ة

**العمر (بالسنة):؟…….**

**الوزن (بالكيلوجرام):؟………**

**الطول (بالسانتي متر)؟……..**

**كم عدد افراد الأسرة؟……**

**الدخل الشهري للأسرة (ريال سعودي):؟.............**

**المستوى التعليمي:**

**-** ابتدائي أو أقل

- متوسط

- ثانوي

- شهادة جامعية أو أعلى

**الحالة الوظيفية:**

- طالب

- موظف

- اعمال حره

- عاطل

**الحالة الاجتماعية:**

**-** أعزب

- متزوج

- مطلق

- أرمل

ا**ذا كان لديك أبناء هل تم تشخيص أحدهم بمرض الأنيميا المنجلية؟**

- لايوجد لدي أبناء

- لم يتم تشخيص أي إبن من ابنائي بالأنيميا المنجلية

- لدي أبناء مصابين وعددهم؟ (….)

**السكن:**

 - مع الأسرة

- أعيش وحيدا في منزل مستقل

**مكان الإقامة:**

 - مدينة

- قرية

- منطقة جبلية

**هل يوجد أمراض مزمنة أخرى غير الأنيميا المنجلية؟ (خيارات متعددة)**

- ارتفاع ضغط الدم

- أمراض القلب

- مرض السكري

- أمراض الدهون

- إلتهاب المفاصل

- أمراض الجهاز الهضمي

- الأمراض النفسية والعصبية

 - أخرى؟ ………

- لايوجد أمراض أخرى.

**-الجزء الأول: يهتم بالعبء المالي الذي تسببه الأنيميا المنجليه**

١- كم المبلغ التقريبي في الشهر الذي يتم صرفه على المسكنات والأدوية غير الوصفية للسيطرة على اعراض الأنيميا المنجلية؟ ……….(المبلغ بالريال السعودي)

٢- كم المبلغ تقريباً في الشهر الذي يتم دفعه من أجل إحضار مقدم رعاية للمنزل بسبب مرض الأنيميا المنجلية؟ ……….(المبلغ بالريال السعودي)

٣- كم المبلغ تقريباً في الشهر الذي يتم دفعه من أجل الحصول على الرعاية الصحية في المستشفى بسبب الأنيميا المنجلية (فحوصات، علاج، تنويم… إلخ)؟ ……….(المبلغ بالريال السعودي)

٤- كم المبلغ تقريباً الذي يتم صرفه من اجل الوصول للمستشفى أو السفر لعلاج الأنيميا المنجلية (بنزين، تذاكر طيران، سكن، مواصلات، أكل…الخ)؟ ……….(المبلغ بالريال السعودي)

**ب-الجزء الثاني: يهتم بتأثير الأنيميا المنجلية على أيام العمل (أو الدراسة):**

٥- كم عدد الأيام في الشهر تقريباً التي تتغيب فيها عن العمل (أو عن الدراسة) لزيارة المستشفى بسبب الأنيميا المنجلية؟ عدد الأيام: ………..

٦- كم عدد الأيام في الشهر تقريباً التي تتغيب فيها عن العمل (أو عن الدراسة) بسبب اللآلام وأعراض الأنيميا المنجلية؟ عدد الأيام: ………..

٧- كم عدد الأيام في الشهر تقريباً التي يتغيب فيها أحد الأقارب ( الوالدين، الأخوان، الأقارب، الأصدقاء ) عن العمل لتقديم الرعاية لك بسبب الأنيميا المنجلية ؟ عدد الأيام: ………

**-الجزء ماقبل الأخير: الجداول التالية تدرس الصحة من جميع الجوانب وتشمل المشاعر والآلام التي تتعرض لها:**

- حدد كم من الوقت خلال الثلاثين يوم الماضية شعرت بالمشاعر التالية:

| **أ ( الصحه النفسيه )** | | | | | |
| --- | --- | --- | --- | --- | --- |
| أبداً | نادراً | قليلاً | غالباً | دائماً |  |
|  |  |  |  |  | ١- خلال الأيّام الثلاثين الماضية، كم غالباً كنت تشعر بأنك متوتر؟ |
|  |  |  |  |  | ٢- خلال الأيّام الثلاثين الماضية، كم غالباً كنت تشعر بأنك يائس؟ |
|  |  |  |  |  | ٣- خلال الأيّام الثلاثين الماضية، كم غالباً كنت تشعر بأنك متململ أو متضايق؟ |
|  |  |  |  |  | ٤- خلال الأيّام الثلاثين الماضية، كم غالباً كنت تشعر بأنك شديد الإكتآب لدرجة أنه لم يعد أي شيء يفرحك؟ |
|  |  |  |  |  | ٥- خلال الأيّام الثلاثين الماضية، كم غالباً كنت تشعر بأنك تتطلب مجهودًا للقيام بأي شيء؟ |
|  |  |  |  |  | ٦- خلال الأيّام الثلاثين الماضية، كم غالباً كنت تشعر بأنك لا قيمة لك؟ |

- حدد مدى إتفاقك مع كل عبارة مما يلي فيما يخص تأثير الأنيميا المنجلية على صحتك البدنية:

| **ب ( الصحة البدنيه )** | | | | | |
| --- | --- | --- | --- | --- | --- |
| معارض بشدة | معارض | محايد | أتفق | أتفق بشدة |  |
|  |  |  |  |  | ١- لستُ راضٍ مطلقاً عن صحتي الجسدية بسبب مرض الأنيميا المنجلية. |
|  |  |  |  |  | ٢- لا أستطيع الحصول على النوم الكافي بسبب الآلام والأعراض التي تسببها الأنيميا المنجلية. |
|  |  |  |  |  | ٣- أنا غير قادر على تأدية نشاطاتي اليومية بكل كفاءه بسبب تأثير الأنيميا المنجلية وأعراضها على صحتي الجسدية. |
|  |  |  |  |  | ٤- أحتاج إلى الأدوية أو الأجهزة الطبية لتأدية أعمالي اليومية بشكل طبيعي. |
|  |  |  |  |  | ٥- تؤثر الأنيميا المنجلية على مقدرتي وإنتظامي في العمل. |
|  |  |  |  |  | ٦- أشعر بصعوبة شديدة في الحصول على عمل أو بيئة تناسب صحتي الجسدية. |

- حدد مدى إتفاقك مع كل عبارة مما يلي فيما يخص الآلام الناتجة بسبب الأنيميا المنجلية:

| **ج ( الآلام )** | | | | | |
| --- | --- | --- | --- | --- | --- |
| معارض بشدة | معارض | محايد | أتفق | أتفق بشدة |  |
|  |  |  |  |  | ١- أنا قلق طوال الوقت حول ما إذا كان الألم سينتهي أم لا. |
|  |  |  |  |  | ٢- أشعر أنني لا أستطيع الاستمرار فيما أشعر فيه من الألم. |
|  |  |  |  |  | ٣- الألم فظيع، وأعتقد أنه لا يمكن أن يتحسن. |
|  |  |  |  |  | ٤- الألم مروع، وأشعر أنه يثقل كاهلي و يتعبني. |
|  |  |  |  |  | ٥- أشعر أنه لا يمكنني التحمل أكثر بعد الآن. |
|  |  |  |  |  | ٦- ليس بوسعي فعل أي شيء للتقليل من حدة الألم. |

- حدد مدى إتفاقك مع كل عبارة مما يلي فيما يخص تأثير الأنيميا المنجلية على علاقاتك الاجتماعية:

| **د ( الحياة الإجتماعية )** | | | | | |
| --- | --- | --- | --- | --- | --- |
| معارض بشدة | معارض | محايد | أتفق | أتفق بشدة |  |
|  |  |  |  |  | ١- أشعر بالرضى التام عن علاقاتي الشخصية. |
|  |  |  |  |  | ٢- أحصل على القدر الكافي من المساعدة والعون ممن هم حولي (الأهل، الأصدقاء، عامة الناس). |
|  |  |  |  |  | ٣- أستطيع تقديم يد العون للآخرين بكل سهولة ورضى ولا تعيقني الأنيميا المنجلية عن ذلك. |
|  |  |  |  |  | ٤- لا تؤثر الانيميا المنجلية على علاقاتي الاجتماعية وتواصلي مع زملائي في مكان العمل (المدرسة، الجامعة، الوظيفة. إلخ ). |
|  |  |  |  |  | ٥- لا تؤثر الأنيميا المنجلية على تواصلي مع عامة الناس في الأماكن العامة (البقالة، السوق ..إلخ). |

**-الجزء الأخير: الجدول الأسئلة التالية تهتم بالعلاقة مع الطبيب المعالج والأدوية المستخدمة والعمليات المجراة:**

| **أ -( العلاقة مع الطبيب المعالج)** | | | | | |
| --- | --- | --- | --- | --- | --- |
| معارض بشدة | معارض | محايد | أتفق | أتفق بشدة |  |
|  |  |  |  |  | ١- طبيبي المعالج للأنيميا المنجلية مهتم بمساعدتي. |
|  |  |  |  |  | ٢- يسأل طبيبي المعالج عن الأعراض التي تسببها الأنيميا المنجلية في كل موعد. |
|  |  |  |  |  | ٣- أشعر بالارتياح عندما أناقش الأعراض التي ظهرت علي مع طبيبي المعالج. |
|  |  |  |  |  | ٤- أثق انه يتم معالجتي ومراقبة مرضي بالشكل الصحيح. |
|  |  |  |  |  | ٥- يفهم طبيبي مدى تأثير مرض الأنيميا المنجلية على حياتي. |
|  |  |  |  |  | ٦- يُعلِمُني طبيبي بأي خيارات علاجية جديدة. |
|  |  |  |  |  | ٧- يناقش معي طبيبي أي أعراض جانبية من الممكن أن تسببها الأدوية التي استخدمها. |
|  |  |  |  |  | ٨- لدي أنا وطبيبي نفس الأهداف للسيطرة على الأنيميا المنجلية وعلاجها. |
|  |  |  |  |  | ٩- أشعر بأن مرضي بالأنيميا المنجلية لا يتم علاجه بالشكل الأفضل. |
|  |  |  |  |  | ١٠- أتجنب رؤية طبيبي ومناقشة الأنيميا المنجلية معه بسبب خوفي. |

**ب- إختر جميع الأدوية المستخدمة حالياً أو تم استخدامها في الماضي لعلاج الأنيميا المنجلية وأعراضها (خيارات متعدده):**

- الهايدروكسي يوريا (كيورميل).

- دواء إل قلوتاماين .

- كريزان ليزوماب (أداكفيو).

- مسكنات الألم التي لاتحتاج لوصفه.

- الأفيونات ( مسكنات الألم القوية والتي تصرف بوصفة طبية ).

- مضادات حيوية.

- مضادات التهاب.

- ادوية لخفض مستوى الحديد.

- حمض الفوليك أو أي معادن أخرى.

- فيتامين د أو غيرها من الفيتامينات.

- نقل دم.

- سوائِل وأملاح.

- تطعيمات.

- يوجد أشياء أخرى ولا أستطيع تذكرها.

- أي أدوية أو مساعِدات طبيه أخرى ( مثل: مساج، إعادة تأهيل، اكسجين منزلي ..إلخ )؟

……….

- لا يأخذ المريض أي أدوية للأنيميا المنجلية.

**ج- هل يوجد أي عمليات جراحية قام بها المريض بسبب الأنيميا المنجلية؟ (خيارات متعدده)**

- عملية إستئصال الطحال.

- عملية إستئصال اللوز.

- عملية إستئصال المرارة.

- عملية تبديل مفصل.

- عملية في العين.

- عملية بسبب مشاكل في الكبد.

- عملية بسبب مشاكل في الكلى.

- أخرى؟ …….

- لم يقم المريض بأي عمليات بسبب الأنيميا المنجلية.
