# Supplementary material for: Clinical, humanistic, and economic burden of sickle cell disease in The Jazan Region, Saudi Arabia
Source: PLoS One. 2026 May 14;21(5):e0348759. doi: 10.1371/journal.pone.0348759 (PMC13175482; doi:10.1371/journal.pone.0348759)
Supplement: S3 File — (DOCX) [file pone.0348759.s003.docx]

**1**. Diagnosis

Have you been diagnosed with sickle cell anemia by a specialist?

- Yes

- No

---

**2**. Personal Information

Gender:

- Male

- Female

Nationality:

-Saudi

-Non-Saudi

Age (year):

Ans______

Weight (Kg):

Ans______

Height (cm):

Ans______

Number of family members:

Ans______

Monthly Family Income (Saudi Riyal):

Ans______

Educational Level:

- Elementary or below

- Intermediate

- High school

- University degree or higher

Employment Status:

- Student

- Employed

- Free business

- Unemployed

Marital Status:

- Single

- Married

- Divorced

- Widower

If you have children, has any of them been diagnosed with sickle cell anemia?

- I do not have children.

- None of my children have been diagnosed.

- Yes, I have children diagnosed with sickle cell anemia. Number of affected children: ______

Living Arrangement:

- With family

- Alone in a separate residence.

Residence Location:

- City

- Village

- Mountain area

Governorate:

- Abu-Arish

- Ahad Almasariha

- Al-Harth

- Al-Daer

- Al-Darb

- Al-Rayth

- Al-Tuwal

- Al-Arrdah

- Al-Aydabi

- Baysh

- Jayzan

- Samtah

- Sabya

- Damad

- Farasan

- Fayfa

- Haroub

Do you have any chronic diseases other than sickle cell anemia?

- Hypertension

- Heart disease

- Diabetes

- Dyslipidemia

- Arthritis

- Gastrointestinal diseases

- Neurological or psychiatric disorders

- Others: ______ (please specify)

**3**. **Financial** and **Time** Burden Related to Sickle Cell Anemia Disease :

1. Approximate monthly expenses on over-the-counter pain relievers and other medications for managing symptoms (SAR):

Ans______

2. Approximate monthly cost of hiring home care services due to sickle cell anemia (SAR):

Ans______

3. Approximate monthly cost of hospital care, including lab tests, treatments, and hospitalizations etc… (SAR):

Ans______

4. Approximate monthly transportation costs to access hospital care (fuel, flights, accommodation, meals, etc.) (SAR):

Ans______

5. Approximate number of work or school days missed monthly due to hospital visits:

Ans______

6. Approximate number of work or school days missed monthly due to pain and other symptoms of sickle cell anemia:

Ans______

7. Approximate number of workdays missed by family members (parents, siblings, relatives, or friends) to care for you:

______

8. Approximate number of social or personal commitments (trips, hobbies, courses, etc.) you had to cancel due to sickle cell anemia:

Ans______

**4**. Impact of Sickle Cell Anemia on **Mental Health**, **Physical Health**, **Pain**, and **Social Life.**

1. Mental Health

| Qs | Response | **Always** | **Most of the time** | **Half of the time** | **Some of the time** | **Never** |
| --- | --- | --- | --- | --- | --- | --- |
| 1.In the past 30 days, how often did you feel stressed? | |  |  |  |  |  |
| 2. In the past 30 days, how often did you feel hopeless? | |  |  |  |  |  |
| 3. In the past 30 days, how often did you feel restless or irritated? | |  |  |  |  |  |
| 4. In the past 30 days, how often did you feel so depressed that nothing could cheer you up? | |  |  |  |  |  |
| 5. In the past 30 days, how often did you find it hard to make an effort to do anything? | |  |  |  |  |  |
| 6. In the past 30 days, how often did you feel worthless? | |  |  |  |  |  |

1. Physical Health

| Qs | Response | **Strongly Agree** | **Agree** | **Neutral** | **Disagree** | **Strongly Disagree** |
| --- | --- | --- | --- | --- | --- | --- |
| 1. I am completely dissatisfied with my physical health due to sickle cell anemia. | |  |  |  |  |  |
| 2. I cannot get enough sleep due to the pain and symptoms of sickle cell anemia. | |  |  |  |  |  |
| 3. I am unable to perform daily activities effectively due to the impact of sickle cell disease. | |  |  |  |  |  |
| 4. I require medication or medical devices to perform my daily tasks. | |  |  |  |  |  |
| 5. Sickle cell anemia affects my ability to work consistently. | |  |  |  |  |  |
| 6. I find it extremely difficult to find suitable employment due to my physical health condition. | |  |  |  |  |  |

1. Pain

| Qs | Response | **Strongly Agree** | **Agree** | **Neutral** | **Disagree** | **Strongly Disagree** |
| --- | --- | --- | --- | --- | --- | --- |
| 1. I am constantly worried about whether the pain will ever end. | |  |  |  |  |  |
| 2. I feel I cannot continue in the pain I experience. | |  |  |  |  |  |
| 3. The pain is terrible, and I believe it cannot improve. | |  |  |  |  |  |
| 4. The pain is overwhelming and exhausting. | |  |  |  |  |  |
| 5. I feel I can no longer bear it. | |  |  |  |  |  |
| 6. I feel powerless to reduce the intensity of the pain. | |  |  |  |  |  |

1. Social Life

| Qs | Response | **Strongly Agree** | **Agree** | **Neutral** | **Disagree** | **Strongly Disagree** |
| --- | --- | --- | --- | --- | --- | --- |
| 1. I am completely satisfied with my personal relationships. | |  |  |  |  |  |
| 2. I receive sufficient help and support from those around me (family, friends, and others). | |  |  |  |  |  |
| 3. I can easily and willingly help others without being hindered by sickle cell anemia. | |  |  |  |  |  |
| 4. Sickle cell anemia does not affect my social relationships with colleagues (at work, school, or university). | |  |  |  |  |  |
| 5. Sickle cell anemia does not affect my interactions with the general public in public spaces (e.g., grocery stores or markets). | |  |  |  |  |  |

**5**. This section addresses the medications used in managing sickle cell anemia and its symptoms, along with the healthcare provider's approach to patient care:

**A. Select all statements that apply to your relationship with your healthcare provider:**

| Qs | Response | **Strongly Agree** | **Agree** | **Neutral** | **Disagree** | **Strongly Disagree** |
| --- | --- | --- | --- | --- | --- | --- |
| 1- My doctor is interested in helping me. | |  |  |  |  |  |
| 2- My doctor asks about symptoms caused by sickle cell anemia during every visit. | |  |  |  |  |  |
| 3- I feel comfortable discussing my symptoms with my doctor. | |  |  |  |  |  |
| 4- I trust that my treatment and disease monitoring are done properly. | |  |  |  |  |  |
| 5- My doctor understands how the disease impacts my life. | |  |  |  |  |  |
| 6- My doctor informs me of new treatment options. | |  |  |  |  |  |
| 7- My doctor discusses the potential side effects of my medications. | |  |  |  |  |  |
| 8- My doctor and I share the same goals in managing the disease. | |  |  |  |  |  |
| 9- I feel my condition is not being managed optimally. | |  |  |  |  |  |
| 10- I avoid seeing my doctor due to fear. | |  |  |  |  |  |

**B. Select all medications you are currently using or have used in the past for sickle cell anemia and its symptoms:**

- Hydroxyurea.

- L-Glutamine.

- Crizanlizumab.

- Over-the-counter pain relievers.

- Prescription opioids.

- Antibiotics.

- Anti-inflammatory drugs.

- Iron-chelating agents.

- Folic acid or other minerals.

- Vitamin D or other vitamins.

- Blood transfusions.

- Fluids and electrolytes.

- Vaccines.

- Other medications but I can’t remember.

- Any other medications or assistance medical devices : ______  (please specify)

- I don’t take any medications for sickle cell disease.

**C. Have you undergone any surgeries related to sickle cell anemia (multiple choices)**

-Spleen removal.

-Tonsils removal.

-Gallbladder removal.

-Joint replacement.

-Surgery in the eye.

-Surgery due to liver problems.

-Surgery due to kidney problems.

-Others: ______  (please specify)

-I didn't have any surgery due to sickle cell disease.
